# Gastrointestinal bleeding caused by rupture of a posterior inferior pancreaticoduodenal artery pseudoaneurysm: A case report

**DOI:** 10.1097/MD.0000000000032123

**Published:** 2022-12-02

**Authors:** JinHua Cui, YaMan Liu, Jian Li

**Affiliations:** a Department of Hepatobiliary Surgery, Affiliated Hospital of Chengde Medical College, Chengde City, Hebei Province, China; b Department of Gynaecology, Affiliated Hospital of Chengde Medical College, Chengde City, Hebei Province, China.

**Keywords:** gastrointestinal bleeding, pancreaticoduodenal aneurysm, visceral artery aneurysm

## Abstract

Gastrointestinal bleeding caused by a ruptured pancreatic pseudoaneurysm is rare, and a pseudoaneurysm of the posterior inferior pancreaticoduodenal artery is especially rare. A 68-year-old man was hospitalized after presenting intermittent black stools and dyspnea accompanied by severe anemia. Angiographic examination revealed that Pseudoaneurysm of the posterior inferior pancreaticoduodenal artery.

**Methods:**

Angiography was performed and revealed a pseudoaneurysm of the posterior inferior pancreaticoduodenal artery. Subsequently, a blood transfusion and endovascular embolization were performed.

**Results:**

The patient’s gastrointestinal bleeding stopped, and the hemoglobin level remained stable. During 1 year of follow-up, the patient remained in a generally good condition.

**Conclusion:**

posterior inferior pancreaticoduodenal artery pseudoaneurysmIt is rare and difficult to diagnose, gastrointestinal bleeding is a serious complication, vascular interventional embolization is effective.

## 1. Introduction

Pancreaticoduodenal aneurysms are relatively rare, accounting for only 2% of all visceral artery aneurysms.^[1,2]^ Pancreaticoduodenal aneurysms can be classified into pseudoaneurysms and true aneurysms. Pseudoaneurysms involve the rupture of the intima and media of the artery, resulting in bulging of the adventitia.^[3]^ Pancreaticoduodenal pseudoaneurysms are even rarer and are associated with pancreatitis, abdominal trauma, septic embolism, iatrogenic injuries, penetrating duodenal ulcers, and malignant tumors.^[4]^ Pancreaticoduodenal pseudoaneurysms are difficult to diagnose and treat. Furthermore, they have complex clinical manifestations and a high incidence of rupture, which can cause abdominal and gastrointestinal bleeding. Gastrointestinal bleeding caused by rupture of a posterior inferior pancreaticoduodenal artery pseudoaneurysm is very rare. This study reports one such case.

## 2. Case report

**Figure 1.**
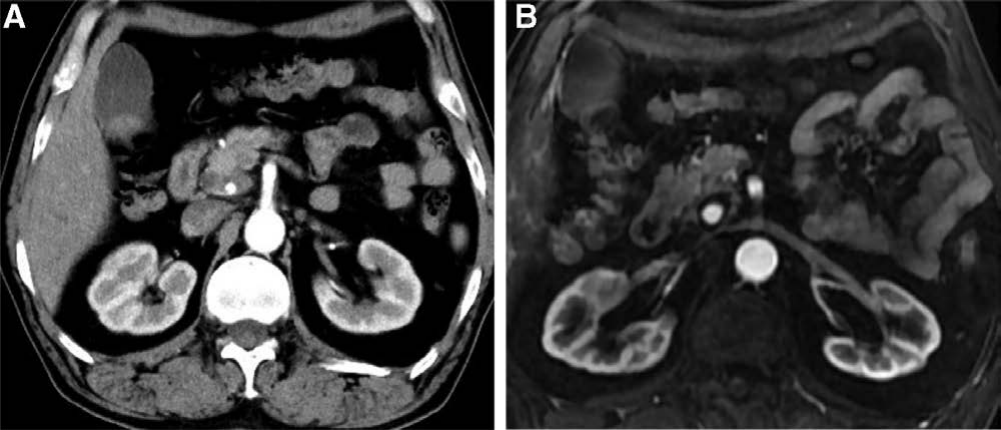
(A) Abdominal computed tomography suggested a possible pancreatic pseudoaneurysm. (B) Abdominal magnetic resonance imaging confirmed a pancreatic pseudoaneurysm.

**Figure 2.**
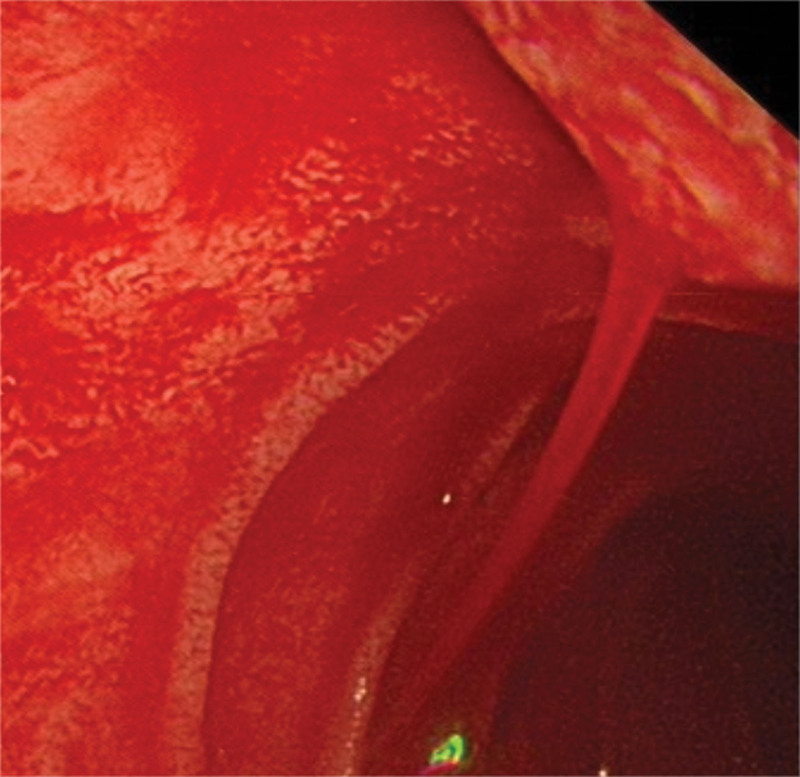
Gastroscopy revealed active bleeding in the duodenal papilla.

The patient was a 68-year-old man who presented to the emergency department because of intermittent melena for 1 month and dyspnea for 1 day. He had a history of hypertension and type 2 diabetes, but no history of gastric ulcer or use of non-steroidal anti-inflammatory drugs. Physical examination revealed no abdominal pain, normal bowel sounds, unheard abdominal sounds, and vascular murmur. On admission, the routine blood test yielded a hemoglobin level of 30 g/L; therefore, he was administered an infusion comprising a suspension of red blood cells (4 U). A subsequent routine blood test indicated a hemoglobin level of 44 g/L. The patient still had severe anemia, so he was again given 4U of red blood cell infusion. A pulmonary computed tomography (CT) examination showed bilateral pleural effusion. An enhanced CT examination of the abdomen revealed a low-density cystic shadow in the pancreatic head area with an obvious small nodular shadow that was considered a pseudoaneurysm (Fig. 1A). Abdominal magnetic resonance imaging revealed a pancreatic pseudoaneurysm (Fig. 1B). The first gastroscopy performed revealed chronic atrophic gastritis with erosion and no bleeding. Colonoscopy revealed no bleeding site. On day 6 after admission, melena reappeared; therefore, the patient underwent emergency gastroscopy, which revealed active bleeding in the duodenal papilla (Fig. 2).

**Figure 3.**
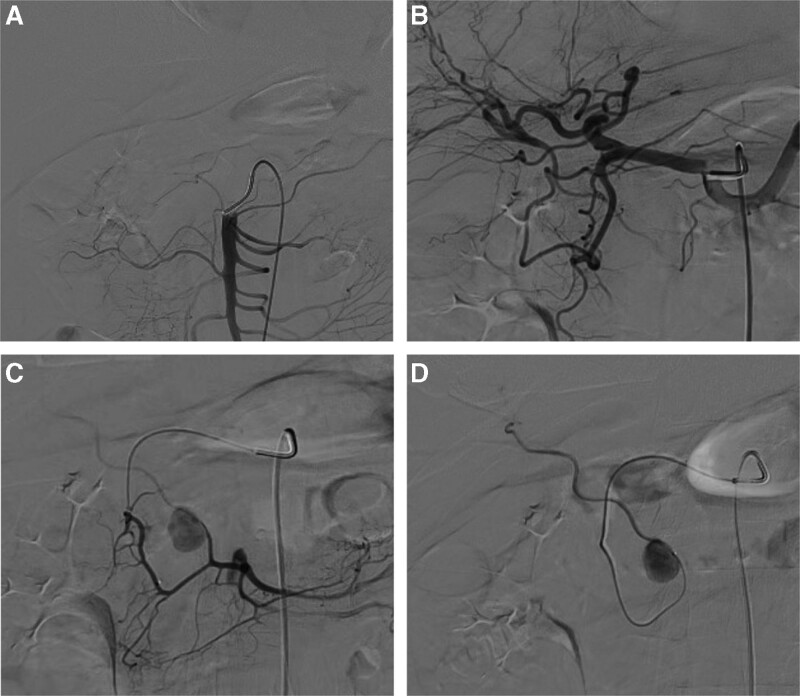
(A) Angiography of the superior mesenteric artery showed no pseudoaneurysm. (B) No pseudoaneurysm was observed using angiography of the celiac trunk. (C) Angiography revealed a pseudoaneurysm of the posterior inferior pancreaticoduodenal artery. The artery supplies blood to the liver. (D) Angiography revealed a pseudoaneurysm as well as blood vessels in the liver.

**Figure 4.**
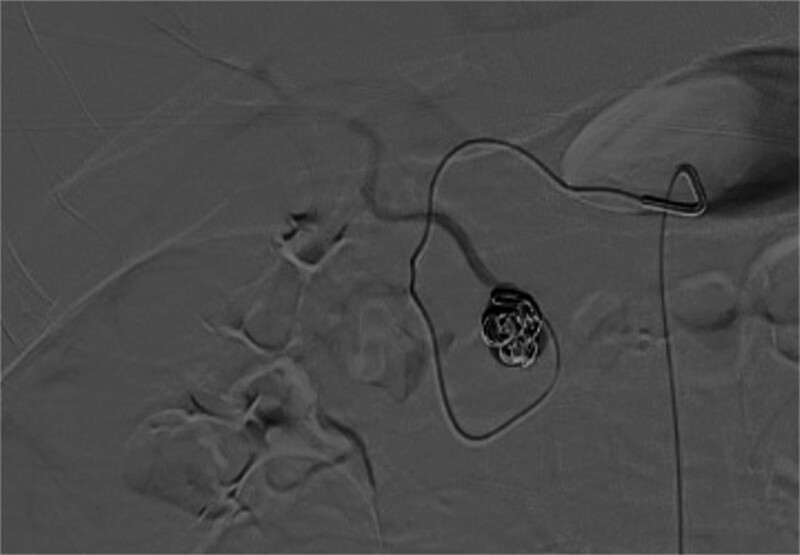
A spring coil was used to fill the pseudoaneurysm. Angiography revealed a pseudoaneurysm with complete tamponade. The blood supply of this artery to the liver was not affected.

Because of the critical condition of the patient, CT angiography of the abdominal artery was not performed. Abdominal angiography showed a punctured right femoral artery. The superior mesenteric artery (Fig. 3A) and celiac trunk (Fig. 3B) were examined after inserting an RH-5F catheter; no pseudoaneurysm or bleeding signs were observed. The microcatheter was superselectively inserted into the gastroduodenal and superior mesenteric arteries; no aneurysms were observed. Additionally, the microcatheter was superselectively inserted into the superior pancreaticoduodenal artery. Angiography showed a pseudoaneurysm in the anterior branch of the inferior pancreaticoduodenal artery and that the artery supplied blood to the liver (Fig. 3C). The microcatheter was pushed forward, and angiography revealed a pseudoaneurysm as well as blood vessels in the liver (Fig. 3D). Therefore, a coil was used to fill the aneurysm. Angiography showed that the aneurysm was well-embolized. The main blood flow was unobstructed, and the blood supply to the liver was also not affected (Fig. 4).

After the postoperative review, the hemoglobin level was maintained at approximately 80 g/L. The patient excreted normal stools after 3 days and did not experience adverse reactions after eating. Therefore, he was discharged. During postoperative follow-up for 1 year, the patient did not experience black stools and was very healthy.

## 3. Discussion

Visceral artery pseudoaneurysms have been associated with pancreatitis, trauma, iatrogenic injuries, penetrating duodenal ulcers, arteritis, and malignancies.^[5]^ Acute pancreatitis is their most common cause.^[6,7]^ However, this patient had no history of or manifestations of acute and chronic pancreatitis. The main complication of a pseudoaneurysm of the inferior pancreaticoduodenal artery is bleeding from aneurysm rupture, with an incidence between 45% and 62%; furthermore, its mortality is high, between 21% and 26%. However, the incidence of pseudoaneurysm rupture is not related to the aneurysm size. In this case, the aneurysm was small, with a diameter less than 1 cm.

Gastrointestinal bleeding caused by pancreatic pseudoaneurysms is often characterized by intermittent bleeding and abdominal pain. The most common clinical symptom is intermittent black stools, followed by hematemesis and hematochezia.^[8]^ The cause of intermittent symptoms may be a blood clot blocking the pancreatic duct during bleeding, resulting in increased pressure in the pancreatic duct and stoppage of the bleeding. When the blood clot dissolves, bleeding occurs again.^[9]^ In addition to intermittent abdominal pain, some patients may experience nausea, vomiting, anorexia, jaundice, and other manifestations. The transaminase level of this patient was ten-times higher than the normal value. An enhanced CT examination of the abdomen showed that the blood vessel where the pseudoaneurysm was located provided blood supply to the liver and that the patient had severe liver damage caused by liver ischemia.

Gastroscopy is not the gold standard technique for diagnosis because bleeding from the pancreatic pseudoaneurysm is intermittent. Only duodenal papillary bleeding can be seen in approximately 30% of cases.^[10]^ However, repeated endoscopy improves the detection rate, especially when patients develop symptoms such as hematochezia or hematemesis. An enhanced CT examination of the abdomen facilitates the diagnosis of the disease. CT angiography and selective angiography often lead to the identification and final diagnosis of peripancreatic aneurysmal disease.

Because of the continuing development and progression, endovascular treatment has become the first-line treatment for patients with pancreatic pseudoaneurysms and stable hemodynamics. Endovascular angiography can accurately diagnose pseudoaneurysms, and embolization treatment can be used for intravascular pseudoaneurysms.^[11–13]^ Studies have shown that, compared to surgical treatment, endovascular treatment can significantly shorten the hospitalization time and reduce trauma for patients. The coil is the most commonly used embolization material for endovascular embolization; however, coil embolization is not the only treatment method. Membrane stents, liquid embolic agents, colloidal bubbles, and thrombin are used for hemostasis.^[14,15]^ However, endovascular embolization is not suitable for all patients with pancreatic pseudoaneurysms. Treatment should be determined according to the size, location, and clinical manifestations of the pseudoaneurysms. Although the success rate of endovascular embolization is very high (79%–100%), the recurrence and bleeding rates are between 18% and 37%.^[13]^ For patients with unstable disease (hemodynamic instability) or failed angiography, surgical treatment of the pseudoaneurysm is recommended.^[11]^ Complications, such as intraoperative bleeding, coil displacement, rebleeding, other vascular embolization, organ ischemic necrosis, and infection, can occur with both treatments. Although open artery ligation, splenectomy, and distal pancreatectomy can provide effective treatment, their risk of perioperative morbidity is greater; furthermore, peripancreatic leakage occurs in 0% to 10% of patients with stable hemodynamics.^[11,13]^ New treatments are being explored, such as endoscopic ultrasound hemostasis, but the treatment effect and prognosis must be further demonstrated.^[16,17]^

Arteriography of the celiac trunk and mesenteric arteries is the most useful diagnostic test for pancreatic pseudoaneurysms, with a sensitivity of 96%.^[10]^ During the treatment of this patient, no pseudoangioma was found on the celiac trunk, superior mesenteric artery, or gastroduodenal artery using angiography. The microcatheter was further inserted into the superior pancreaticoduodenal artery during angiography to identify a pseudoaneurysm located in the posterior branch of the inferior pancreaticoduodenal artery. At the same time, it was found that the blood supply of the vessels in the liver was sufficient, and the aneurysm was embolized. Angiography showed that the aneurysm was well-embolized and the blood supply to the liver was not blocked. This serves as a reminder that it is necessary to perform angiography at multiple sites and further enter the small branch vessels. During surgery, embolization of the pseudoaneurysm was mainly used to Embolic pseudoaneurysm and not minimize the blood supply to the organs.

## 4. Conclusions

A pancreatic pseudoaneurysm is a rare disease with a high mortality rate, especially when it ruptures and bleeds. Therefore, for patients with obscure gastrointestinal bleeding, the possibility of a pancreatic pseudoaneurysm should be considered. An endoscopic examination, enhanced CT examination of the abdomen, CT angiography, and abdominal angiography can help diagnose the disease. Endovascular therapy is the most important treatment option.

## Acknowledgments

We would like to thank Editage (www.editage.com) for English language editing.

## Author contributions

**Writing—original draft:** Cui JinHua.

**Writing—review and editing:** Liu YaMan and LiJian.

## References

[1] Stanley JC Zelenock GB. Splanchnic artery aneurysms. In: Rutherford RB (ed). Vascular surgery. 4th ed. Philadelphia: WB Saunders Co, 1995:1124e39.

[2] Moore E Matthews MR Minion DJ. Surgical management of peripancreatic arterial aneurysms. J Vasc Surg. 2004;40:247–53. 15297817 10.1016/j.jvs.2004.03.045

[3] Dallara H Habboushe J. Spontaneous inferior pancreaticoduodenal artery pseudoaneurysm rupture. Intern Emerg Med. 2017;12:1319–21. 28280981 10.1007/s11739-017-1641-9

[4] Xu QD Gu SG Liang JH. Inferior pancreaticoduodenal artery pseudoaneurysm in a patient with calculous cholecystitis: a case report. World J Clin Cases. 2019;7:2851–6. 31616702 10.12998/wjcc.v7.i18.2851 PMC6789396

[5] Williamson JM Cook JL Jackson JE. Infective aneurysm of the inferior pancreaticoduodenal artery. Ann R Coll Surg Engl. 2001;93:e87–8. 10.1308/147870811X590991 PMC5827065 21929894

[6] Tan J-H Zhou L Cao R-C. Identification of risk factors for pancreatic pseudocysts formation, intervention and recurrence: a 15-year retrospective analysis in a tertiary hospital in China. BMC Gastroenterol. 2018;18:143. 30285639 10.1186/s12876-018-0874-z PMC6167814

[7] Bergert H Hinterseher I Kersting S. Management and outcome of hemorrhage due to arterial pseudoaneurysms in pancreatitis. Surgery. 2005;137:323–8. 15746787 10.1016/j.surg.2004.10.009

[8] Suzuki K Tachi Y Ito S. Endovascular management of ruptured pancreaticoduodenal artery aneurysms associated with celiac axis stenosis. Cardiovasc Intervent Radiol. 2008;31:1082–7. 18414944 10.1007/s00270-008-9343-3

[9] Cui H-Y Jiang C-H Dong J. Hemosuccus pancreaticus caused by gastroduodenal artery pseudoaneurysm associated with chronic pancreatitis: a case report and review of literature. World J Clin Cases. 2021;9:236–44. 33511191 10.12998/wjcc.v9.i1.236 PMC7809673

[10] Regina de Oliveira RS Gabriela Leopoldino S Luiz Fernando R. Embolisation of branches of the superior mesenteric artery in the treatment of haemosuccus pancreaticus. BMJ Case Rep. 2019;12:e229110. 10.1136/bcr-2018-229110 PMC6506096 31068351

[11] Wang Luke L Bauman Zachary M. Hemosuccus pancreaticus: a rare bleeding pseudoaneurysm of the inferior pancreaticoduodenal artery treated with embolization. Case Rep Surg. 2018;2018:2354169. 30245902 10.1155/2018/2354169 PMC6139223

[12] Zyromski Nicholas J Vieira C Stecker M. Improved outcomes in postoperative and pancreatitis-related visceral pseudoaneurysms. J Gastrointest Surg. 2007;11:50–5. 17390186 10.1007/s11605-006-0038-2

[13] Peynircioğlu B Ali Devrim K İdilman İlkay S. Intrapancreatic pseudoaneurysm causing massive gastrointestinal hemorrhage and chronic pancreatitis. Turk J Gastroenterol. 2015;26:270–3. 26006205 10.5152/tjg.2015.6548

[14] Sagar S Soundarajan R Gupta P. Efficacy of endovascular embolization of arterial pseudoaneurysms in pancreatitis: a systematic review and meta-analysis. Pancreatology. 2021;21:46–58. 33303372 10.1016/j.pan.2020.11.017

[15] Williams M Alderson D Virjee J. CT-guided percutaneous thrombin injection for treatment of an inferior pancreaticoduodenal artery pseudoaneurysm. Cardiovasc Intervent Radiol. 2006;29:669–71. 16604412 10.1007/s00270-004-0274-3

[16] Bennett JD. Continuing professional development. Evidence-based radiology problems transcatheter embolization of rectal laceration: December 2003--November 2004. Can Assoc Radiol J. 2003;54:272–6; quiz 276. quiz276. 14689799

[17] Jeffers K Majumder S Santhi Swaroop V. EUS-guided pancreatic pseudoaneurysm therapy: better to be lucky than good. Gastrointest Endosc. 2018;87:1155–6. 29024704 10.1016/j.gie.2017.09.043

